# Living in the sun’s shadow: Skin cancer and albinism in Senegal

**DOI:** 10.1016/j.jdin.2026.04.015

**Published:** 2026-04-27

**Authors:** Roberto Mazzetto, Lorenzo Cipriani, Thiam Astou, Tatiana Pedrazzi, Anna Belloni Fortina, Stefano Piaserico

**Affiliations:** a Dermatology Unit, Department of Medicine (DIMED), University of Padua, Padua, Italy; b Unit of Occupational Health and Industrial Hygiene, Department of Medical Surgical Specialties, Radiological Sciences and Public Health, University of Brescia, Brescia, Italy; c Unité de Dermatologie-Vénéréologie, Université Cheikh Anta Diop de Dakar, Dakar, Senegal; d Department of Women and Child’s Health (SDB) University of Padua, Pediatric Dermatology Regional Center, Padua, Italy

**Keywords:** actinic keratoses, oculocutaneous albinism, photoprotection, skin cancer, Sub-Saharan Africa

*To the Editor:* Oculocutaneous albinism (OCA) is a genetic disorder characterized by reduced or absent melanin production.^1^ To date, 21 genes have been associated with albinism, defining 3 main categories: OCA, ocular albinism (OA), and syndromic forms.^2^ The prevalence is highest in African populations (1:4000) compared with Europeans (1:10,000-1:15,000),^1^ and individuals with albinism frequently develop actinic keratoses (AKs), precursors of squamous cell carcinoma (SCC), following prolonged ultraviolet (UV) exposure.^3^^,^^4^

Based on membership data from the National Association of Albinos of Senegal (ANAS), approximately 100 individuals with albinism are currently registered in the Thiès and Dakar regions. The project was submitted to the local health authorities, who granted authorization to ANAS, which facilitated the recruitment of 61 individuals (61%) who agreed to undergo dermatological examination (33 males and 28 females) between 26 October and 6 November 2024. Clinical data were collected from questionnaires and medical records. Suspected skin cancers were referred by ANAS to surgical care providers for excision, and histopathological examination confirmed both the diagnosis and complete removal of the lesions (Table I).

**Table I:** Prevalence of skin cancers and actinic keratoses (AKs) by age group, residential setting, and sunscreen use

Group	Category	Number of patients (%)	Number of patients with AKs (%)	OR (95% CI)	Number of patients with skin cancers (%)	OR (95% CI)	Total number of skin cancers
Age group	<18 y	16 (26.2)	6 (37.5)	Ref	0	Ref	0
	≥18 y	45 (73.8)	32 (71.1)	4.1 (1.2-13.6)	4 (8.9)	1.1 (1-1.2)	7
Residence	City	36 (59.0)	20 (55.6)	Ref	2 (5.6)	/	2
	Village	25 (41.0)	18 (72.0)	2.1 (0.7-6.1)	2 (8.0)	/	5
Sunscreen use	Yes	37 (60.7)	21 (56.8)	Ref	2 (5.4)	/	2
	No	24 (39.3)	17 (70.8)	1.8 (0.6-5.5)	2 (8.3)	/	5

Values are expressed as percentages within each subgroup. Odds ratios (ORs) with corresponding 95% confidence intervals (95% CI) are reported when applicable.

*AKs*, Actinic keratoses; *Ref*, reference.

During dermatologic examinations, 62.3% (38/61) of participants presented with at least one AK in photo-exposed areas (face, arms, and neck), and skin cancers, namely SCC, were diagnosed in 6.6% (4/61) of participants, all affecting the facial region. AK prevalence was higher among participants who did not use sunscreen (70.8%) than among those who did (56.8%). However, regular sunscreen use was reported by 66.7% of urban residents (24/36), compared with only 52% of rural residents (13/25). In the subgroup of 16 patients younger than 18 years, AKs were present in 37.5%, and the mean number of lesions was 7.4 (SD 4.4), with a maximum of 15 lesions in a single individual. Fig 1 shows a representative patient with multiple AKs on sun-exposed areas, and additional examples are provided in the supplemental material (Supplementary Fig 1 to 5, available via Mendeley at https://data.mendeley.com/datasets/kw8j94m64c/1).

**Fig 1:**
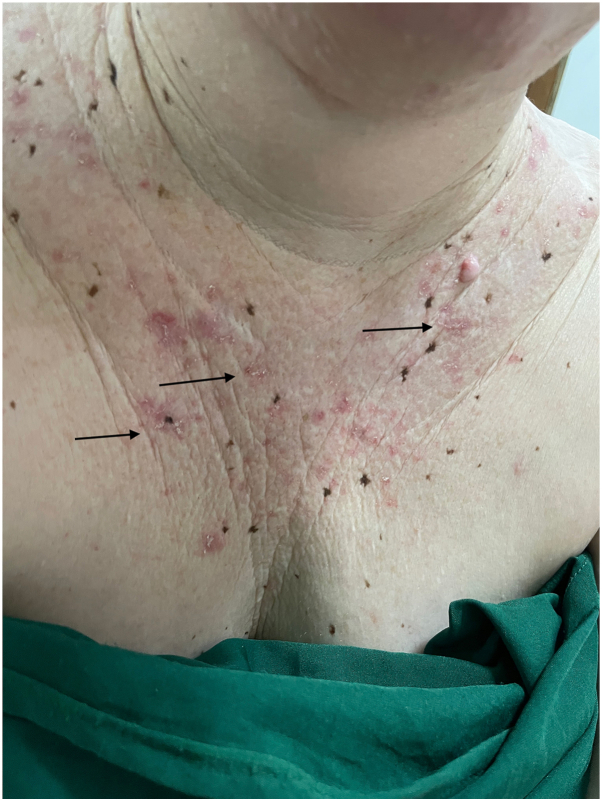
Thirty-year-old woman and multiple actinic keratoses (AKs) on décolleté. The most evident lesions are indicated with *arrows*, although numerous AKs are diffusely present.

Furthermore, we compared our data with findings from a 1995 study conducted in Tanzania, a country with similar geographic conditions.^5^ In that study, AKs were reported in 42% of patients, whereas the prevalence in our cohort was significantly higher (62% vs 42%, *P* < .01). In a more recent study from the Democratic Republic of Congo, only 11.5% of patients with albinism reported regular photoprotection, and the prevalence of skin cancer was 29%.^4^ This difference may reflect the lower use of photoprotection, along with the inclusion in our study of only histologically confirmed skin cancers.

Limitations include recruitment through convenience rather than systematic sampling and the relatively small sample size, which may limit generalizability.

This study shows that UV-induced preneoplastic lesions (AKs) are frequent among individuals with albinism in Senegal, particularly in rural areas. Our findings underscore the importance of photoprotection in reducing UV-related skin disease and call for its promotion as a global public health priority.

Finally, we would like to recognize Silvia Lovera, a key member of the team, whose dedication and commitment were instrumental in organizing and overseeing the activities in Senegal.

## Conflicts of interest

Fortina has been a consultant for Almirall, Amgen, Sanofi Genzyme, Pfizer, AbbVie, Leo Pharma, Unifarco, Novartis and Eli Lilly. Piaserico has been a consultant and/or speaker for Abbvie, Almirall, Celgene, Janssen, Leo-pharma, Eli Lilly, Merck Sharp & Dohme, Novartis, Pfizer, Sandoz, and UCB. Mazzetto, Cipriani, Thiam A, and Pedrazzi have no conflicts of interest.
